# Chemotherapy in Pancreatic Cancer: A Systematic Review

**DOI:** 10.3390/medicina54030048

**Published:** 2018-07-11

**Authors:** Leva Hajatdoost, Keyvan Sedaghat, Erin J. Walker, Jackson Thomas, Sam Kosari

**Affiliations:** 1 Department of Pharmaceutical Sciences, Baha’i Institute for Higher Education (BIHE), Tehran, Iran; leva.hajatdoost@bihe.org (L.H.); keyvan.sedaghat@bihe.org (K.S.); 2 Discipline of Pharmacy, Faculty of Health, University of Canberra, Bruce, Canberra 2617 ACT, Australia; Erin.Walker@canberra.edu.au (E.J.W.); Jackson.Thomas@canberra.edu.au (J.T.)

**Keywords:** chemotherapy, pancreatic cancer, first-line treatment, second-line treatment, survival

## Abstract

*Background and Aim*: Pancreatic cancer is one of the most fatal cancers. Cytotoxic chemotherapy remains the mainstream treatment for unresectable pancreatic cancer. This systematic review evaluated and compared the overall survival (OS) and progression-free survival (PFS) outcomes obtained from recent phase 2 and 3 clinical trials of pancreatic cancer chemotherapy. *Materials and methods*: Thirty-two studies were included and compared based on chemotherapy agents or combinations used. Additionally, outcomes of first-line versus second-line chemotherapy in pancreatic cancer were compared. *Results*: In studies that investigated the treatments in adjuvant settings, the highest OS reported was for S-1 in patients, who received prior surgical resection (46.5 months). In neoadjuvant settings, the combination of gemcitabine, docetaxel, and capecitabine prior to the surgical resection had promising outcomes (OS of 32.5 months). In non-adjuvant settings, the highest OS reported was for the combination of temsirolimus plus bevacizumab (34.0 months). Amongst studies that investigated second-line treatment, the highest OS reported was for the combination of gemcitabine plus cisplatin (35.5 months), then temsirolimus plus bevacizumab (34.0 months). *Conclusions*: There is a need to develop further strategies besides chemotherapy to improve the outcomes in pancreatic cancer treatment. Future studies should consider surgical interventions, combination chemotherapy, and individualized second-line treatment based on the prior chemotherapy.

## 1. Introduction

Pancreatic cancer is one of the most fatal cancers and a leading cause of cancer-related mortality. The five-year survival rate for pancreatic cancer in the United States was reported at 8%, which was the lowest among many other common types of cancer [1]. Only about 20% of pancreatic cancers are resectable, thus surgical resection is an essential component of any curative treatment modality for this cancer where it is possible [2]. A recent study investigated the usefulness of radiofrequency ablation in the resection of tumors, and found this technique aided separation and dissection of the tumor from blood vessels; although this study was very small (*n* = 6), the results were promising [3]. More than 80% of pancreatic cancers are locally advanced or metastatic at the time of diagnosis [4]. Targeted and immune therapies have not been successful to date [5]. Adjuvant chemotherapy with radiation or alone have been used widely in an attempt to improve outcomes [2]. Cytotoxic chemotherapy has remained as the mainstream treatment for unresectable pancreatic cancer [5]. Since the approval of gemcitabine by Food and Drug Administration in 1997 [5], several cytotoxic agents have been used in monotherapy or combination therapy in phase 2 and 3 trials worldwide, with no significant improvement in prolongation of overall survival (OS) or progression-free survival (PFS) [6]. A recent systematic review by Rahib et al. [6] explored pancreatic cancer phase 3 trials conducted between 1992 and 2015 and critically analyzed the association between phase 2 and phase 3 outcomes. This study identified that 32 phase 3 studies, examining 27 agents or combinations in 13,675 chemotherapy-naive patients with advanced pancreatic cancer, resulted in three agents or combinations that are considered clinically meaningful. Ten studies proceeded to phase 3 despite phase 2 negative results. Eight studies proceeded to phase 3 without a prior phase 2 trial and one study included a third arm in the phase 3 without a prior phase 2 trial. Only 6 trials out of 32 phase 3 studies met the primary endpoint, but showed only a modest improvement in survival of patients with pancreatic cancer [6]. Overall, the findings indicated the lack of a systematic approach in designing clinical trials that consequently resulted in the lack of progress in pancreatic cancer treatment.

This systematic review investigated recent phase 2 and 3 trials of chemotherapy agents or combinations (including combination with radiation therapy) in patients with pancreatic cancer with or without prior chemotherapy, reported between 1 January 2015 and 1 April 2017, and compared the outcomes in terms of OS or PFS.

## 2. Methods

This systematic review was conducted in accordance with Preferred reporting items for systematic reviews and meta-analyses (PRISMA) guidelines [7]. Bibliographic searches were carried out in PubMed for recent clinical trial studies published between 1 January 2015 and 1 April 2017. The search comprised the terms pancreatic cancer chemotherapy, pancreatic cancer treatment, and pancreatic cancer radiotherapy. Additional manual searches in reference lists of the relevant articles were also conducted. Only phase 2 and phase 3 clinical trials that examined chemotherapy or chemotherapy plus radiotherapy were selected. The population of interest was composed of individuals with any type or stage of pancreatic cancer, metastatic pancreatic cancer, or pancreatic neuroendocrine tumors with or without prior chemotherapy. Phase 1 studies, studies about diseases other than pancreatic cancer, and studies that investigated any therapy other than chemotherapy or chemoradiotherapy were excluded. The primary outcome measure was overall survival (OS). The secondary outcome was progression-free survival (PFS). Studies that measured neither OS nor PFS were excluded.

### 2.1. Data Extraction

Two reviewers independently assessed the titles and abstracts of articles to determine trial inclusion. Information from the full texts using a predefined data extraction sheet was extracted. Disagreements were resolved by discussion. Extracted study details were study characteristics (first author, year of the publication, study design), study population (number of patients in each arm), treatment (agents or combination of chemotherapy with or without radiation therapy, chemotherapy drug dosage, and duration), presence of prior treatment, OS, and PFS.

### 2.2. Quality Assessment

The methodological quality of studies was assessed using a previously published assessment tool (the Effective Public Health Practice Project Quality Assessment Tool (EPHPP)) [8]. Quality appraisal was conducted by two researchers, with differences resolved through discussion. Criteria used to assess the methodological quality of trials included selection bias, study design (including randomization), control for confounders, blinding data collection methods, and the reporting of withdrawals and dropouts. Options for each question were “yes”, “no”, or “can’t tell”. Studies that received “no” or “can’t tell” to both questions were given a weak rating for quality of execution for that criterion. Studies were reported as strong (no weak ratings); moderate (one weak rating); or weak (two or more weak ratings).

### 2.3. Data Synthesis

Trials were categorized based on chemotherapy agents or combinations to allow for comparison between endpoint outcomes in trials where the same drugs were used with different combinations. Trials were also categorized based on the presence of prior treatment to investigate the potential impact of prior treatment on the response to second-line trial agents or combinations. OS and PFS were used to compare the outcomes.

## 3. Results

**Figure 1:**
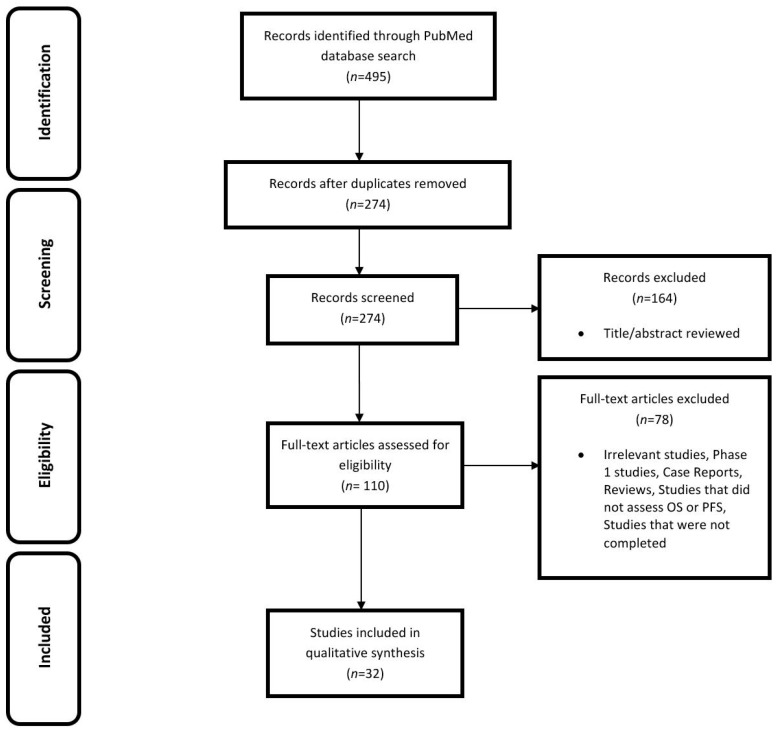
PRISMA flow diagram. OS—overall survival; PFS—progression-free survival.

The initial search of the literature using keywords according to the inclusion criteria retrieved 495 records. Following the removal of 221 duplicate studies, 274 potentially relevant studies were identified. After screening these 274 studies more carefully based on their titles and abstracts, 164 articles were excluded. The remaining full-text articles were studied and assessed for eligibility; irrelevant articles, phase 1 studies, reviews, case reports, studies that investigated any therapy other than chemotherapy or chemoradiotherapy, and those that did not assess OS or PFS were also excluded. Finally, 32 clinical trials (phase 2 and 3) reported between 1 January 2015 and 1 April 2017 fulfilled the inclusion criteria. The language of all articles was English. Figure 1 shows the flow diagram for study selection.

From 32 eligible studies, 21 were phase 2, 7 were phase 3, and 4 were phase 1/2 (only their phase 2 information was considered). The characteristics of studies are summarized in Supplementary Table S1. Studies were categorized based on chemotherapy agents or combinations that were used to allow for comparison between endpoint outcomes resulted from different treatment strategies.

### 3.1. Quality Assessment

Quality assessment of the studies was conducted as described in the “Methods” section. From 32 studies, 2 were rated as strong, 13 were rated as moderate, and 17 were rated as weak (Supplementary Table S2).

### 3.2. Classification of Trials Based on Drugs Used

#### 3.2.1. Gemcitabine with Other Drugs

##### Gemcitabine and Tegafur/Gimeracil/Oteracil (S-1)

**Figure 2:**
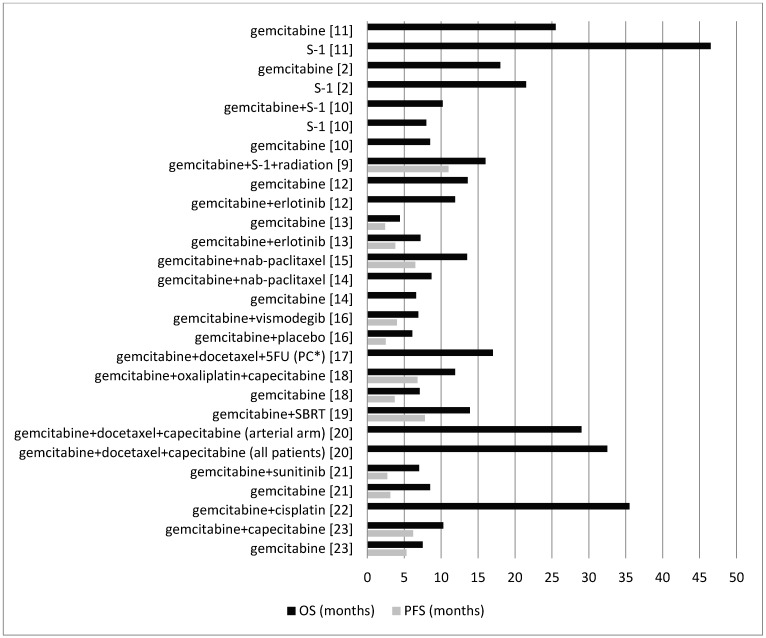
Comparison between the OS and PFS for different combinations of gemcitabine with other agents. PC*: pancreatic cancer; 5FU: 5-fluorouracil.

Four clinical trials, two phase 3, one phase 2, and one phase 1/2, assessed gemcitabine and S-1, alone or in combination [2,9,10,11]. Uesaka et al. [11] assessed gemcitabine or S-1 in patients with invasive ductal pancreatic carcinoma, stage I–III, with no local residual or microscopic residual tumor, and those with resected pancreatic cancer with no history of chemotherapy or radiotherapy within the past three years. Median PFS was not assessed. Median OS was 25.5 months in the gemcitabine group and 46.5 months in the S-1 group. Shimoda et al. [2] assessed gemcitabine or S-1 in patients following the surgical resection of pancreatic cancer. Median PFS was not assessed. Median OS was 18.0 months in the gemcitabine group and 21.5 months in the S-1 group. The combination of gemcitabine and S-1 was assessed by Imaoka et al. [10]. In that study, gemcitabine plus S-1, S-1 alone, and gemcitabine alone were trialed in elderly patients (≥70 years) with unresectable pancreatic cancer. Median PFS was not assessed. Median OS was 10.2 months in the gemcitabine plus S-1 group, 8.0 months in the S-1 only group, and 8.5 months in the gemcitabine only group. Goji et al. [9] assessed fixed-dose rate gemcitabine and S-1 with a total radiation dose delivered concurrently in patients with unresectable pancreatic cancer confined to the pancreatic region with no prior treatment for pancreatic cancer. Median PFS was 11.0 months and median OS was 16.0 months (Figure 2).

##### Gemcitabine and Erlotinib

Two clinical trials, one phase 3 and one phase 2, assessed the combination of gemcitabine and erlotinib [12,13]. Hammel et al. [12], in the first randomization, assessed gemcitabine alone and gemcitabine plus erlotinib in patients with locally advanced pancreatic cancer. In the second randomization, they assessed gemcitabine alone, gemcitabine plus erlotinib, and radiation therapy plus capecitabine in patients with progression-free disease after four months. In this study, patients had no prior chemotherapy or radiation therapy. Median PFS was not assessed. From the date of the first randomization, median OS in the gemcitabine group was 13.6 months and in the gemcitabine plus erlotinib group was 11.9 months. Wang et al. [13] assessed gemcitabine or gemcitabine plus erlotinib in chemotherapy-naive metastatic pancreatic cancer patients. Median PFS in the gemcitabine plus erlotinib group was 3.8 months and in the gemcitabine group was 2.4 months. Patients with epidermal growth factor receptor (EGFR) mutations in the gemcitabine group had similar median PFS, but in the gemcitabine plus erlotinib group had a significantly longer PFS (5.9 months versus 2.4 months). Median OS in the gemcitabine plus erlotinib group was 7.2 months and in the gemcitabine group was 4.4 months. Consistent with OS data, patients with EGFR mutations in the gemcitabine group had a similar median OS, but in the gemcitabine plus erlotinib group had a significantly longer OS (8.7 months versus 6.0 months) (Figure 2).

##### Gemcitabine and Nab-Paclitaxel

Two clinical trials, one phase 1/2 and one phase 3, assessed the combination of gemcitabine and nab-paclitaxel [14,15]. Ueno et al. [15] assessed nab-paclitaxel followed by gemcitabine in Japanese patients with metastatic pancreatic cancer (no prior therapy excluding surgery). Median PFS and median OS were 6.5 and 13.5 months, respectively. Goldstein et al. [14] assessed gemcitabine plus nab-paclitaxel or gemcitabine alone in patients with metastatic pancreatic cancer with no prior chemotherapy for the metastatic disease. Median PFS was not assessed. Median OS in the gemcitabine plus nab-paclitaxel group was 8.7 months and in the gemcitabine group was 6.6 months (Figure 2).

##### Gemcitabine and Vismodegib

Catenacci et al. [16] assessed the combination of gemcitabine plus vismodegib or gemcitabine plus placebo in patients with pancreatic cancer not amenable to surgical resection who had received no prior therapy for the metastatic disease. Median PFS was 4.0 months in the gemcitabine plus vismodegib group and 2.5 months in the gemcitabine plus placebo group. Median OS was 6.9 months in the gemcitabine plus vismodegib group and 6.1 months in the gemcitabine plus placebo group (Figure 2).

##### Gemcitabine, Docetaxel, and 5-Fluorouracil (5FU)

Cho et al. [17] treated patients with pancreaticobiliary cancers after a curative-intent surgical resection (with no prior chemotherapy or radiation therapy) with two cycles of gemcitabine plus docetaxel followed by 5FU-based chemoradiation. Two cycles of gemcitabine and docetaxel were administered after completion of chemoradiation. Median PFS was not assessed. Median OS was 17.0 months in the patients with pancreatic cancer and 23.0 months in the patients with resected biliary tract cancer (Figure 2).

##### Gemcitabine, Oxaliplatin, and Capecitabine

Petrioli et al. [18] compared the combination of gemcitabine, oxaliplatin, and capecitabine versus gemcitabine alone in patients with metastatic pancreatic cancer with no prior chemotherapy. Median PFS was 6.8 months in the group treated with the combination regimen and 3.7 months in the gemcitabine alone group. Moreover, median OS was 11.9 months in the combination regimen group and 7.1 months in the gemcitabine alone group (Figure 2).

##### Gemcitabine and Stereotactic Body Radiotherapy (SBRT)

Herman et al. [19] assessed gemcitabine treatment followed by a one-week break, followed by SBRT and then continued gemcitabine therapy in patients with locally advanced pancreatic cancer with no prior abdominal radiotherapy and no more than three doses of gemcitabine before SBRT. Median PFS was 7.8 months and median OS was 13.9 months (Figure 2).

##### Gemcitabine, Docetaxel, and Capecitabine

Sherman et al. [20] evaluated neoadjuvant gemcitabine, docetaxel, and capecitabine in patients with pancreatic adenocarcinoma presenting with locally advanced, unresectable disease because of arterial or extensive venous involvement. Patients in both arterial and venous arms were treated with gemcitabine, docetaxel, and capecitabine. Then, only patients in the arterial arm received additional treatment with gemcitabine and capecitabine/radiation therapy following the initial treatment. PFS was not assessed. Median OS was 32.5 months in all patients, 29.0 months in patients in the arterial arm, and more than 42.0 months in the venous arm, with at least three patients remaining disease-free beyond 34.0 months. Patients in the venous arm survived significantly longer than those in the arterial arm (Figure 2).

##### Gemcitabine and Sunitinib

Bergmann et al. [21] assessed gemcitabine versus a combination of gemcitabine plus sunitinib in patients with locally advanced, unresectable, or metastatic pancreatic ductal adenocarcinoma without previous chemotherapy. Median PFS was 3.1 months in the gemcitabine group and 2.7 months in the gemcitabine plus sunitinib group. Median OS was 8.5 months in the gemcitabine group and 7.01 months in the gemcitabine plus sunitinib group (Figure 2).

##### Gemcitabine and Cisplatin

Postlewait et al. [22] assessed the combination of gemcitabine and cisplatin in patients with resected pancreatic adenocarcinoma with previous gemcitabine therapy. PFS was not assessed. Median OS was 35.5 months (Figure 2).

##### Gemcitabine and Capecitabine

Lee et al. [23] examined the combination of gemcitabine plus capecitabine or gemcitabine alone in patients with advanced pancreatic cancer without prior chemotherapy. Median PFS was 6.2 months in the gemcitabine plus capecitabine group and 5.3 months in the gemcitabine group. Median OS was 10.3 months in the gemcitabine plus capecitabine group and 7.5 months in the gemcitabine group (Figure 2).

#### 3.2.2. Capecitabine with Other Drugs (Except Gemcitabine)

##### Capecitabine and Lapatinib

**Figure 3:**
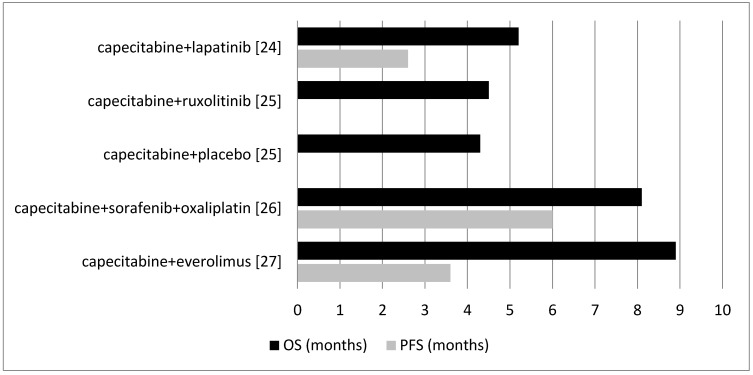
Comparison between the OS and PFS for different combinations of capecitabine with other agents except gemcitabine.

Wu et al. [24] assessed the combination of capecitabine and lapatinib in patients with metastatic unresectable pancreatic cancer whose disease had progressed on first-line gemcitabine-based therapy. Median PFS was 2.6 months and median OS was 5.2 months (Figure 3).

##### Capecitabine and Ruxolitinib

Hurwitz et al. [25] reported the combination of capecitabine plus ruxolitinib or capecitabine plus placebo in patients with metastatic pancreatic cancer who had treatment failure with prior gemcitabine treatment. Median PFS was not assessed. Median OS was 4.5 months in the capecitabine plus ruxolitinib group and 4.3 months in the capecitabine plus placebo group (Figure 3).

##### Capecitabine, Sorafenib, and Oxaliplatin

Makielski et al. [26] assessed sorafenib along with oxaliplatin followed by capecitabine in patients who had just one previous chemotherapy for their pancreatic adenocarcinoma. Median PFS was 6.0 months and median OS was 8.1 months (Figure 3).

##### Capecitabine and Everolimus

Kordes et al. [27] examined the combination of capecitabine and everolimus in patients with advanced adenocarcinoma of the pancreas in addition to patients with prior chemotherapy in the adjuvant setting or for metastatic disease. Median PFS was 3.6 months and median OS was 8.9 months (Figure 3).

#### 3.2.3. Other Drug Combinations

##### Modified FOLFIRINOX (Irinotecan and Bolus 5-fluorouracil Reduced by 25%)

**Figure 4:**
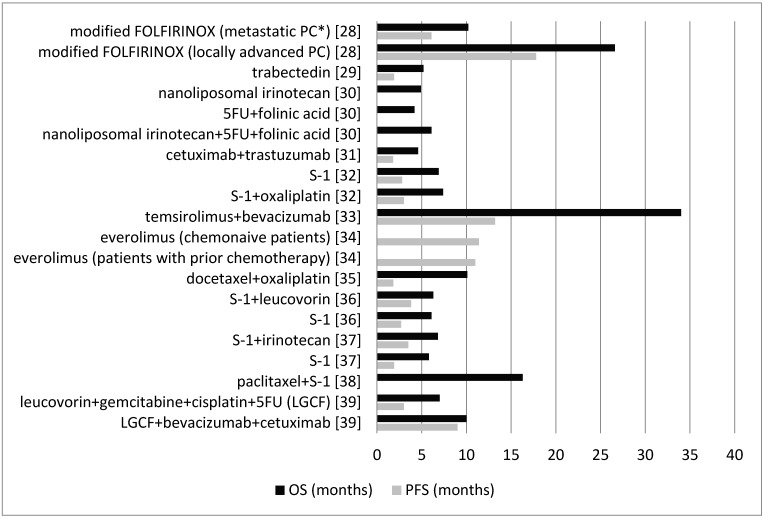
Comparison between the OS and PFS for the other drug combinations (except gemcitabine and capecitabine). *PC: pancreatic cancer. LGCF—leucovorin, gemcitabine, cisplatin, and 5FU.

Stein et al. [28] investigated modified FOLFIRINOX in patients with untreated metastatic or locally advanced pancreatic cancer. Included study patients did not receive any prior chemotherapy except that prior adjuvant chemotherapy or radiotherapy for resected pancreatic adenocarcinoma was allowed only if the treatment occurred more than six months prior to the registration. Median PFS was 6.1 months in the metastatic pancreatic cancer group and 17.8 months in the locally advanced pancreatic cancer group. Median OS was 10.2 months in the metastatic pancreatic cancer group and 26.6 months in the locally advanced pancreatic cancer group (Figure 4).

##### Trabectedin

Belli et al. [29] examined trabectedin in patients with metastatic pancreatic adenocarcinoma after unsuccessful gemcitabine-based first-line chemotherapy. Median PFS was 1.9 months and median OS was 5.2 months (Figure 4).

##### Nanoliposomal Irinotecan, 5FU, and Folinic Acid

Wang-Gillam et al. [30] assessed the effect of nanoliposomal irinotecan monotherapy; or the combination of 5FU and folinic acid; and in the third arm, examined the combination of nanoliposomal irinotecan with 5FU and folinic acid in patients with metastatic pancreatic ductal adenocarcinoma previously treated with gemcitabine-based therapy. Median PFS was not assessed. Median OS was 6.1 months in the nanoliposomal irinotecan plus 5FU and folinic acid group, 4.2 months in the 5FU and folinic acid group, and 4.9 months in the nanoliposomal irinotecan monotherapy group (Figure 4).

##### Cetuximab and Trastuzumab

Assenat et al. [31] assessed the combination of cetuximab and trastuzumab in patients with advanced pancreatic cancer after failure of the first-line gemcitabine-based chemotherapy. Median PFS was 1.8 months and median OS was 4.6 months (Figure 4).

##### S-1 and Oxaliplatin

Ohkawa et al. [32] examined S-1 alone or a combination of S-1 plus oxaliplatin in patients with progressive disease following first-line treatment with a gemcitabine-based therapy. Median PFS was 2.8 months in the S-1 group and 3.0 months in the S-1 plus oxaliplatin group. Median OS was 6.9 months in the S-1 group and 7.4 months in the S-1 plus oxaliplatin group (Figure 4).

##### Temsirolimus and Bevacizumab

Hobday et al. [33] assessed the combination of temsirolimus and bevacizumab in patients with well or moderately differentiated pancreatic neuroendocrine tumors with progressive disease. Median PFS was 13.2 months and median OS was 34.0 months (Figure 4).

##### Everolimus

Lombard-Bohas et al. [34] examined everolimus or placebo in patients with advanced, progressive, and low- or intermediate-grade pancreatic neuroendocrine tumors with no prior chemotherapy, immunotherapy, or radiotherapy within four weeks before randomization. Median PFS in the chemo-naive patients was 11.4 months with everolimus and 5.4 months with placebo. Median PFS in the patients with prior chemotherapy was 11.0 months with everolimus and 3.2 months with placebo. Median OS was not assessed (Figure 4).

##### Docetaxel and Oxaliplatin

Ettrich et al. [35] assessed the combination of docetaxel and oxaliplatin in patients with chemo-refractory advanced pancreatic ductal adenocarcinoma with previous chemotherapy experience (including gemcitabine as the first-line therapy for metastatic pancreatic cancer). Median PFS was 1.82 months and median OS was 10.1 months (Figure 4).

##### S-1 and Leucovorin

Ueno et al. [36] compared the combination of S-1 plus leucovorin with S-1 monotherapy in patients with gemcitabine-refractory advanced pancreatic cancer. Median PFS was 3.8 months in the S-1 plus leucovorin group and 2.7 months in the S-1 group. Median OS was 6.3 months in the S-1 plus leucovorin group and 6.1 months in the S-1 group (Figure 4).

##### S-1 and Irinotecan

Ioka et al. [37] compared the combination of S-1 plus irinotecan with S-1 alone in patients with gemcitabine-refractory pancreatic cancer. Median PFS was 3.5 months in the S-1 plus irinotecan group and 1.9 months in the S-1 group. Median OS was 6.8 months in the S-1 plus irinotecan group and 5.8 months in the S-1 group (Figure 4).

##### Paclitaxel and S-1

Satoi et al. [38] assessed the combination of intravenous and intraperitoneal paclitaxel plus S-1 in chemotherapy-naive pancreatic ductal adenocarcinoma patients with peritoneal metastasis. PFS was not assessed. Median OS was 16.3 months (Figure 4).

##### Leucovorin, Gemcitabine, Cisplatin, 5FU, Bevacizumab, and Cetuximab

Tai et al. [39] compared the combination of leucovorin, gemcitabine, cisplatin, and 5FU (LGCF) with the combination of LGCF plus bevacizumab and cetuximab in patients with histologically or cytologically confirmed pancreatic adenocarcinoma (locally advanced or metastatic pancreatic cancer) that was not amenable to curative treatment with surgery or had been documented or suspected of metastases to extra-pancreatic sites. Patients with no prior chemotherapy were included. Median PFS was 3.0 months in the LGCF group and 9.0 months in the LGCF plus bevacizumab and cetuximab group. Median OS was 7.0 months in the LGCF group and 10.0 months in the LGCF plus bevacizumab and cetuximab group (Figure 4).

### 3.3. Chemotherapy and Radiotherapy

Four trials [9,17,19,20] assessed combinations of chemotherapy and radiotherapy in patients with pancreatic cancer. Treatments and endpoint outcomes are summarized in Table 1.

### 3.4. First-Line versus Second-Line Chemotherapy

Seventeen trials included in this systematic review limited the treatment to patients with no prior chemotherapy or radiotherapy (Table 2) and 14 trials examined the second-line treatment in patients who had prior chemotherapy (Table 3). In trials that examined the first-line therapies, most of the treatments (15 out of 17 studies) were based on gemcitabine therapy, alone or in combination with other agents. In trials that investigated the second-line treatment, patients in the majority of studies (13 out of 14 studies) also had prior treatments with gemcitabine.

## 4. Discussion

This systematic review investigated the recent phase 2 and 3 clinical trials conducted between 2015 and 2017 that assessed OS or PFS of different chemotherapy combinations for pancreatic cancer treatment. All 32 included studies assessed OS, PFS, or both as their primary or secondary outcomes. Trials were categorized and compared based on the treatment combinations, inclusion of radiation therapy, and being a first-line or second-line treatment. It is not possible to directly compare the endpoint outcomes obtained from different studies, given that treatments were conducted in different patients and settings. However, reviewing the trends of the use of chemotherapeutic agents or combinations in recent trials can reveal a pattern that may provide a more targeted approach toward the choice of first- or second-line therapies in future pancreatic cancer treatment and research.

Gemcitabine has been the standard first-line treatment for pancreatic cancer for many years. A recent systematic review in 2016 [6] revealed that the OS following the treatment with gemcitabine has increased from a median of 5.5 months (in studies conducted prior to 2000) to a median of 8.1 months (in studies conducted since 2000). This is likely to be due to better supportive care measures and the ability to tolerate subsequent lines of therapy. Sixteen different studies included in this systematic review assessed gemcitabine alone or compared gemcitabine with other combinations. From the included studies, the study by Uesaka et al. [11] had the highest OS (25.5 months) with adjuvant gemcitabine. Of the examined gemcitabine combinations, the combination of adjuvant gemcitabine and cisplatin resulted in the highest OS (35.5 months) [22]. In the neoadjuvant settings, the combination of neoadjuvant gemcitabine, docetaxel, and capecitabine in a subgroup of patients with adenocarcinoma of the pancreas and extensive venous involvement resulted in the highest OS (more than 42.0 months) [20]; and the combination of gemcitabine and vismodegib had the lowest OS (6.9 months) [16]. Median PFS was only assessed in a few studies and ranged from 2.4 to 11.0 months.

Adjuvant S-1, in the study by Uesaka et al. [11], had the highest OS (46.5 months), which, in that study, was superior to gemcitabine alone and was also higher than the other gemcitabine combinations in other studies. The high OS reported in this study may be because of the fact that the participants underwent surgical resection of the pancreatic cancer prior to the treatment. Uesaka et al. reported that grade 3 or 4 adverse events frequently experienced in the S-1 group were abnormal levels of leukocytes, neutrophils, hemoglobin and platelets, as well as fatigue, anorexia, and diarrhea. S-1 was well tolerated in the adjuvant setting and could be administered with a higher relative dose-intensity than gemcitabine, even after pancreatectomy [11]. However, in non-adjuvant settings, S-1 alone [10,37] and its combinations, including, S-1 plus gemcitabine [10], S-1 plus oxaliplatin [32], S-1 plus leucovorin [36], and S-1 plus irinotecan [37], resulted in poor OS outcomes; in contrast, some studies found more promising results, such as the combination of S-1 with gemcitabine plus radiation [9] and S-1 plus paclitaxel [38]. The discrepancies in results may be because of differences in the study population in terms of progression of the disease.

Four different combinations of capecitabine were examined in the included studies. The combination of capecitabine and everolimus had the highest OS (8.9 months) [27] in this category and capecitabine plus placebo had the lowest OS (4.3 months) [25]. Similarly, the combination of capecitabine and ruxolitinib had a low OS (4.5 months) [25]. Median PFS results were not promising. The combination of capecitabine and lapatinib had the lowest PFS (2.6 months) [24]. Although this combination is increasingly being used in breast cancer treatments [40], the outcomes in pancreatic cancer were quite poor. The combination of capecitabine, sorafenib, and oxaliplatin had the highest PFS (6.0 months) [26] among capecitabine combinations. Overall, capecitabine and its combination drug regimens (except with gemcitabine) did not show promising results.

This systematic review investigated 12 other different drug combinations that were examined in recent trials. Nine studies assessed both OS and PFS, one study only reported PFS and two studies only reported OS. From these, temsirolimus plus bevacizumab had the highest OS (34.0 months) (PFS: 13.2 months) [33] and 5FU plus folinic acid had the lowest OS (4.2 months) [30]. Modified FOLFIRINOX for locally advanced pancreatic cancer patients also had promising survival outcomes (OS: 26.6 months; PFS: 17.8 months) (modified FOLFIRINOX was more efficient in locally advanced pancreatic cancer patients than in patients with untreated metastatic pancreatic cancer) [28].

In studies that investigated the treatments in adjuvant settings, the highest OS reported was for S-1 in patients who received prior surgical resection (46.5 months) [11]. In neoadjuvant settings, the combination of gemcitabine, docetaxel, and capecitabine prior to the surgical resection had promising outcomes in terms of OS (32.5 months for all patients, 29.0 months for the arterial arm patients, and more than 42.0 months for the venous arm patients) [20]. In non-adjuvant settings, the highest OS reported was for the combination of temsirolimus plus bevacizumab (34.0 months) [33].

Studies that combined radiation therapy with chemotherapy [9,17,19,20] resulted in median OS between 13.9 and 29.0 months, suggesting that radiation therapy can be also considered in the treatment regimen. It should be noted that radiation therapy is not feasible in all patients because of anatomical restrictions and disease involvement.

### First-Line versus Second-Line Therapy

Since the development of resistance to gemcitabine, which was the standard treatment for pancreatic cancer for many years, several more aggressive regimens were developed and tested to overcome resistance mechanisms by the cancer cells [5]. The standard first-line therapy in pancreatic cancer is a matter of debate. Most guidelines consider FOLFIRINOX and nab-paclitaxel plus gemcitabine as the accepted treatment for the first-line therapy of pancreatic cancer in fit patients with a trend towards better survival outcomes for FOLFIRINOX [5,41]. For second-line chemotherapy following the failure of first-line therapy, there is not enough evidence for a standard therapy [5,42].

Among the studies that investigated second-line treatment in patients with prior chemotherapy, the highest OS reported was for the combination of gemcitabine plus cisplatin (35.5 months) [22]. The participants of the study had prior treatment with gemcitabine. The combination of gemcitabine and cisplatin in those patients resulted in grade 3/4 toxicity that occurred in 59% of the patients; neutropenia was the most common (in 41% of patients), which was determined to be acceptable by the authors [22]. The combination of temsirolimus plus bevacizumab had the second highest OS (34.0 months) [33]. This combination was examined in patients who received prior treatment with two or less prior cytotoxic chemotherapy regimens, interferon, radio labeled somatostatin analog, prior octreotide and/or continued octreotide at a stable dose, or prior hepatic arterial therapies for liver metastasis. The most common grade 3 to 4 adverse effects were hypertension, fatigue, lymphopenia, hyperglycemia, and thrombocytopenia. Thirty percent of patients discontinued study treatment because of adverse effects or refusal. There was grade 2 to 3 fatigue and mucositis in more than half of these patients [33]. As expected, the OS of most of those with no prior chemotherapy was higher than those with prior chemotherapy. It has been suggested that a personalized plan for second-line treatment based on response to the first-line treatment be developed [43]; however, this requires obtaining further evidence through future trials focused on the second-line treatment. The outcomes from recent phase 2 and 3 studies included in this systematic review suggest that addition of cisplatin to gemcitabine for patients who received gemcitabine as the first-line treatment or combination of temsirolimus plus bevacizumab as the second-line treatment may provide better survival outcomes; however, this needs further investigation.

## 5. Conclusions

Despite many recent phase 2 and 3 trials, the treatment and survival outcomes of pancreatic cancer remain poor. There is a need to develop further strategies besides chemotherapy to improve the outcomes in pancreatic cancer treatment. It is recommended that future studies should consider surgical interventions where possible besides chemo or radiation therapy, use combination therapy rather than using a single agent, conduct separate sub-analysis of survival outcomes based on the level of disease progression at the start of the treatment, and consider any prior treatment and provide individualized treatment strategy based on the prior chemotherapy to address the gap in the knowledge of appropriate second-line therapies.

## Figures and Tables

**Table 1:** Comparison between the overall survival (OS) and progression-free survival (PFS) for trials with chemotherapy and radiotherapy treatment strategies in pancreatic cancer patients.

Reference	Treatment	Overall Survival (OS)	Progression-Free Survival (PFS)
Goji et al. [9]	Fixed-dose-rate gemcitabine and S-1 (both orally). A total radiation dose of 50.4 Gy was delivered concurrently.	16.0 months	11.0 months
Cho et al. [17]	Gemcitabine and docetaxel followed by 5-fluorouracil (5FU)-based chemoradiation. Four weeks after completing chemoradiation, two cycles of gemcitabine and docetaxel were administered.	17.0 months in patients with pancreatic cancer and 23.0 months in patients with resected biliary tract cancer.	NA
Herman et al. [19]	Gemcitabine followed by a one-week break and stereotactic body radiotherapy, then followed by gemcitabine.	13.9 months	7.8 months
Sherman et al. [20]	Patients in the arterial arm were treated with initial chemotherapy (gemcitabine, docetaxel, and capecitabine) followed by gemcitabine and capecitabine/radiation therapy.	29.0 months	NA

**Table 2:** Comparison between the OS and PFS of trials with chemotherapeutic agents in patients with no prior chemotherapy.

Reference	Treatment	Overall Survival (OS)	Progression-Free Survival (PFS)
Uesaka et al. [11]	1- Gemcitabine 2- S-1	1- 25.5 months 2- 46.5 months	NA
Hammel et al. [12]	1- Gemcitabine alone 2- Gemcitabine plus erlotinib	1- 13.6 months 2- 11.9 months	NA
Ueno et al. [15]	Nab-paclitaxel followed by gemcitabine.	13.5 months	6.5 months
Imaoka et al. [10]	1- Gemcitabine plus S-1 2- S-1 alone 3- Gemcitabine alone	1- 10.2 months 2- 8.0 months 3- 8.5 months	NA
Catenacci et al. [16]	1- Vismodegib plus gemcitabine 2- Gemcitabine plus placebo	1- 6.9 months 2- 6.1 months	1- 4.0 months 2- 2.5 months
Goji et al. [9]	Fixed-dose-rate gemcitabine and S-1 (both orally). A total radiation dose of 50.4 Gy was delivered concurrently.	16.0 months	11.0 months
Wang et al. [13]	1- Gemcitabine 2- Gemcitabine plus erlotinib	1- 4.4 months 2- 7.2 months	1- 2.4 months 2- 3.8 months
Shimoda et al. [2]	1- Adjuvant chemotherapy with S-1 after resection of pancreatic cancer 2- Adjuvant chemotherapy with gemcitabine after resection of pancreatic cancer	1- 21.5 months 2- 18.0 months	NA
Cho et al. [17]	Two cycles of gemcitabine and docetaxel followed by 5FU-based chemoradiation. Four weeks after completing chemoradiation, two cycles of gemcitabine and docetaxel were administered.	17.0 months for patients with pancreatic cancer and 23.0 months for patients with resected biliary tract cancer.	NA
Goldstein et al. [14]	1- Nab-paclitaxel + gemcitabine 2- Gemcitabine alone	1- 8.7 months 2- 6.6 months	NA
Petrioli et al. [18]	1- GEMOXEL arm: combination of single-agent Gemcitabine, Oxaliplatin, and Capecitabine 2- Gemcitabine alone	1- 11.9 months 2- 7.1 months	1- 6.8 months 2- 3.7 months
Sherman et al. [20]	1- Neoadjuvant gemcitabine, docetaxel, and capecitabine followed by gemcitabine and capecitabine/radiation therapy in patients in the arterial arm 2- Neoadjuvant gemcitabine, docetaxel, and capecitabine in patients in the venous arm	1- 29.0 months 2- More than 42.0 months 32.5 months for all 45 patients.	NA
Lombard-Bohas et al. [34]	Everolimus or placebo	NA	11.4 months with everolimus and 5.4 months with placebo.
Bergmann et al. [21]	1- Gemcitabine 2- Gemcitabine plus sunitinib	1- 8.5 months 2- 7.01 months	1- 3.1 months 2- 2.7 months
Tai et al. [39]	1- Leucovorin, gemcitabine, cisplatin, and fluorouracil 2- Leucovorin, gemcitabine, cisplatin, and fluorouracil plus bevacizumab and cetuximab.	1- 7.0 months 2- 10.0 months	1- 3.0 months 2- 9.0 months
Lee et al. [23]	1- Gemcitabine plus capecitabine 2- Gemcitabine alone	1- 10.3 months 2- 7.5 months	1- 6.2 months 2- 5.3 months
Satoi et al. [38]	Intravenous and intraperitoneal paclitaxel plus S-1.	16.3 months	NA

NA—not available.

**Table 3:** Comparison between the OS and PFS of trials with chemotherapeutic agents in patients with prior chemotherapy experience.

Reference	Prior Chemotherapy Drugs (Treatment Failure)	Second-Line Treatment	Overall Survival (OS)	Progression-Free Survival (PFS)
Belli et al. [29]	Gemcitabine	Trabectedin	5.2 months	1.9 months
Wang-Gillam et al. [30]	Gemcitabine	1- Nanoliposomal irinotecan plus fluorouracil and folinic acid 2- Fluorouracil and folinic acid 3- Nanoliposomal irinotecan monotherapy	1- 6.1 months 2- 4.2 months 3- 4.9 months	NA
Wu et al. [24]	Gemcitabine	Lapatinib and capecitabine	5.2 months	2.6 months
Hurwitz et al. [25]	Gemcitabine	1- Ruxolitinib plus capecitabine 2- Placebo plus capecitabine	1- 4.5 months 2- 4.3 months	NA
Makielski et al. [26]	Prior gemcitabine-containing therapy (*n* = 15 out of 24). Prior pancreatectomies (*n* = 2 out of 24). Previous pancreatic radiotherapy (*n* = 3 out of 24).	Sorafenib, oxaliplatin, and two days of high-dose capecitabine	8.1 months	6.0 months
Assenat et al. [31]	Gemcitabine	Cetuximab and trastuzumab	4.6 months	1.8 months
Ohkawa et al. [32]	Gemcitabine	1- S-1 monotherapy 2- S-1 plus oxaliplatin	1- 6.9 months 2- 7.4 months	1- 2.8 months 2- 3.0 months
Kordes et al. [27]	Prior gemcitabine-based chemotherapy (*n* = 18 out of 31). Palliative setting (*n* = 11 out of 31). Adjuvant (*n* = 6 out of 31; 5 patients with chemotherapy free interval <6months and 1 as part of neoadjuvant chemoradiotherapy).	Capecitabine and everolimus	8.9 months	3.6 months
Herman et al. [19]	Gemcitabine	Gemcitabine and stereotactic body radiotherapy	13.9 months	7.8 months
Lombard-Bohas et al. [34]	Two or less prior cytotoxic chemotherapy regimens, interferon, radio labeled somatostatin analog, prior octreotide and/or continued octreotide at a stable dose, or prior hepatic arterial therapies for liver metastasis	Temsirolimus and bevacizumab	34.0 months	13.2 months
Ettrich et al. [35]	Gemcitabine	Docetaxel and oxaliplatin	10.1 months	1.82 months
Ueno et al. [36]	Gemcitabine	1- S-1 plus leucovorin 2- S-1 monotherapy	1- 6.3 months 2- 6.1 months	1- 3.8 months 2- 2.7 months
Postlewait et al. [22]	Gemcitabine	Gemcitabine and cisplatin	35.5 months	NA
Ioka et al. [37]	Gemcitabine	1- S-1 plus irinotecan 2- S-1 alone	1- 6.8 months 2- 5.8 months	1- 3.5 months 2- 1.9 months

## Supplementary Materials

The following is available online at http://www.mdpi.com/1010-660X/54/3/48/s1: Supplementary Table S1, Supplementary Table S2.
