# Supplementary material for: Chemotherapy in Pancreatic Cancer: A Systematic Review
Source: Medicina (Kaunas). 2018 Jul 11;54(3):48. doi: 10.3390/medicina54030048 (PMC6122094; doi:10.3390/medicina54030048)
Supplement: Supplementary file 1 [file medicina-54-00048-s001.pdf]

Supplementary Table 1. Characteristics of the included studies.

Number	Clinical trial/Phase/Study design	Reference	Year	Target population	Treatment(s)	Primary endpoint of efficacy/ Secondary outcomes	Patients on treatment(s),n	Median OS	Median PFS
1	NCT00690300, DocOx (AIO-PK0106)<br><br>Phase II<br><br>An open label, multicenter, single arm, study.	Ettrich *et al.* *[35]*	2016	Patients with chemo-refractory advanced pancreatic ductal adenocarcinoma with previous chemotherapy experience (for example gemcitabine as first line therapy for metastatic pancreatic cancer).	**75 mg/m²** **docetaxel** for 60 minutes on day 1 and **80 mg/m²** **oxaliplatin** for 120 minutes on day 2 in 21-day cycles. The treatment period was scheduled for up to 8 cycles.	Primary endpoint: tumor response according to RECIST 1.0. Secondary endpoints: PFS, OS, safety/toxicity, quality of life and clinical benefit.	44	10.1 months	1.82 months
2	JapicCTI-111554<br><br>Phase II<br><br>An open-labeled, multicenter, randomized study.	Ueno *et al.* *[36]*	2016	Patients with gemcitabine-refractory advanced pancreatic cancer (first-line treatment was with gemcitabine).	Patients were randomly assigned to receive **S-1 at 40, 50 or 60 mg** according to body surface area **plus 25 mg leucovorin**, both given orally twice	Primary endpoint: PFS<br><br>Secondary endpoints: OS, response rate (RR), disease	140 (69 patients in **S-1 plus leucovorin group** and 71 patients in **S-1 group)**.	6.3 months for **S-1 plus leucovorin group** and 6.1 months for **S-1 group**.	3.8 months for **S-1 plus leucovorin group** and 2.7 months for **S-1 group**.

					daily for 1 week, repeated every 2 weeks; or **S-1 monotherapy** at the same dose as the S-1-leucovorin group for 4 weeks, repeated every 6 weeks.	control rate (DCR), duration of response, time to treatment failure (TTF), time to progression (TTP), dose intensity, and adverse events.			
3	Phase II<br><br>A retrospective, tow-armed study.	Tai *et al.* *[39]*	2016	Patients with histological or cytologically confirmed pancreatic adenocarcinoma (locally advanced or metastatic pancreatic cancer) not amenable to curative treatment with surgery or had been documented or suspected of metastases to extra-pancreatic sites. Patients with no prior	**Conventional group** received **conventional therapy (leucovorin, gemcitabine, cisplatin, and fluorouracil)** in two week intervals for 12 cycles. **Targeted group** received **conventional therapy plus bevacizumab and cetuximab** in each cycle (two week	Primary endpoint: OS<br><br>Secondary endpoints: PFS and the safety profiles of the combined therapy.	59 (28 patients in **conventional group** and 31 patients in **targeted group**).	7 months for **conventional group** and 10 months for **targeted group**.<br><br>OS for patients **≤60 years old**: 5 months for **conventional group** and 12 months for **targeted group.**<br><br>There was no difference	3 months for **conventional group** and 9 months for **targeted group**.<br><br>PFS for patients **≤60 years old**: 3 months for **conventional group** and 10 months

				chemotherapy were included.	intervals for 12 cycles). Conventional treatment arm: **1000 mg/ m²** **gemcitabine, 50 mg/ m²** **cisplatin** for the days 1, 8 and 15. The treatment interval for each cycle was 21 days. Targeted group: gemcitabine and cisplatin on day 1 **similar to the conventional group, 5 mg/kg bevacizumab (at 8 mg/kg in the first cycle) with 200 mg/ m²** **cetuximab (at 350 mg/ m² in the first cycle)** and did not receive gemcitabine on the day 8 or 15.			between OS of two treatment groups in patients **>60 years old**.	for **targeted group.**<br><br>PFS for patients **>60 years old**: 3 months for **conventional group** and 7 months for **targeted group**.
4	JASPAC 01 Phase III Randomized, open-label, multicentre,	Uesaka *et al.* [11]	2016	Patients with histologically proven invasive ductal	**Gemcitabine (1000 mg/m(2),** intravenously	Primary endpoint: OS in the two	377 (190 to the **gemcitabine group**	25.5 months for **gemcitabine group**; 46.5	NA

	non-inferiority trial.			carcinoma of the pancreas, pathologically documented stage I-III, and no local residual or microscopic residual tumor, and were aged 20 years or older were eligible. Patients with resected pancreatic cancer with no history of chemotherapy or radiotherapy within the past 3 years.	administered on days 1, 8, and 15, every 4 weeks [one cycle], for up to six cycles) or **S-1** (**40 mg, 50 mg, or 60 mg** according to body-surface area, orally administered twice a day for 28 days followed by a 14 day rest, every 6 weeks [one cycle], for up to four cycles).	treatment groups.	and 187 to the **S-1 group**).	months for **S-1 group.**	
5	LAP07<br><br>Phase III<br><br>An international, open-label, randomized trial.	Hammel *et al.* [12]	2016	Patients with locally advanced pancreatic cancer. In second randomization: patients with progression-free disease after 4 months. Patients had no prior chemotherapy or radiation therapy.	**1000 mg/m2** weekly of **gemcitabine alone** or **1000 mg/m2** of **gemcitabine plus 100 mg/d** of **erlotinib**.<br>In second randomization: patients received 2 months of the **same chemotherapy** or underwent **chemoradiot**	Primary endpoint:<br>OS from the date of the first randomization.<br><br>Secondary outcomes: effect of erlotinib and quality assuranc	449 (442 in first randomization and 269 in second randomization) .	13.6 months from the date of the first randomization for the 223 patients receiving **gemcitabine**. 11.9 months for the 219 patients receiving **gemcitabine plus erlotinib**.	NA

					**herapy** (54 Gy plus **capecitabine**).	e of radiotherapy on OS, PFS of gemcitabine-erlotinib and erlotinib maintenance with gemcitabine alone at the second randomization, and toxic effects.		OS from the date of the first randomization was not significantly different between chemotherapy at 16.5 months and chemoradiotherapy at 15.2 months.	
6	Phase II<br><br>An open label single arm multi-institutional study.	Stein *et al.* [28]	2016	Patients with untreated metastatic pancreatic cancer (MPC) or locally advanced pancreatic cancer (LAPC).<br>No prior therapy of any type for advanced disease was allowed.<br>Prior adjuvant chemotherapy or radiotherapy	**Modified FOLFIRINOX (irinotecan and bolus 5-fluorouracil reduced by 25%).**	Primary endpoint: response rate (RR), median PFS and median OS of modified FOLFIRINOX.<br><br>Adverse events were compared with full-dose	75 (31 with LAPC, 44 with MPC).	In **metastatic pancreatic cancer group**: 10.2 months.<br>In **locally advanced pancreatic cancer group**: 26.6 months.	In **metastatic pancreatic cancer group**: 6.1 months.<br>In **locally advanced pancreatic cancer group**: 17.8 months.

				for resected pancreatic adenocarcinoma was allowed if more than 6 months had elapsed since completion of prior therapy and registration.		FOLFIRINOX.			
7	JapicCTI-121987<br><br>Phase I/II<br><br>A non-randomized, open-label, multicenter trial.	Ueno *et al.* [15]	2016	Japanese patients with metastatic pancreatic cancer. Patients with no prior therapy excluding surgery.	**125 mg/m(2) nab-paclitaxel** followed by **1000 mg/m(2) gemcitabine** on day 1, 8, and 15 every 4 weeks.	Overall response rate according to Response Evaluation Criteria In Solid Tumors (RECIST) in phase II.	34	13.5 months	6.5 months
8	GEST study<br><br>Phase III<br><br>Subgroup analysis of a randomized trial.	Imaoka *et al.* [10]	2016	Elderly patients (≥ 70 years) with un-resectable pancreatic cancer.	**Gemcitabine plus S-1 (GS)** (**1000 mg/m2** IV gemcitabine on days 1 and 8 plus S-1 orally twice daily), or **S-1 alone** (at a dose calculated according to the body surface area (BSA)) or	Primary endpoint: OS<br><br>Secondary endpoints: PFS, objective response rate and safety.	261 (90 for gemcitabine plus S-1 group, 85 for S-1 group, and 86 for gemcitabine group).	10.2 months for **gemcitabine plus S-1 group**, 8.0 months for **S-1 group** and 8.5 months for **gemcitabine group**.	6.9 months in the **gemcitabine plus S-1 group**, 4.2 months in the **S-1 group** and 4.5 months in the **gemcita**

					**gemcitabine alone** (1000 **mg/m2** on days 1, 8, and 15 of a 28-day cycle).				**bine group**.
9	Phase II<br><br>A single-center, prospective, single-arm study.	Belli *et al.* [29]	2016	Patients with metastatic pancreatic adenocarcinoma (gemcitabine-resistant disease) after gemcitabine-based first-line chemotherapy.	**Trabectedin 1.3 mg/m(2)** as a 3-h intravenous continuous infusion every 3 weeks for a maximum of 6 months.	Primary endpoint: PFS rate at 6 months (PFS-6).<br><br>Secondary outcome: to identify inflammatory biomarkers predictive for response to trabectedin.	25	5.2 months (range 1.1-24.3).	1.9 months (range 0.8-7.4).
10	NAPOLI-1 (NCT01494506)<br><br>Phase III<br><br>A global, randomized, open-label trial.	Wang-Gillam *et al.* [30]	2016	Eligible patients with metastatic pancreatic ductal adenocarcinoma previously treated with gemcitabine-based therapy.	**Nanoliposomal irinotecan monotherapy** (**120 mg/m(2)** every 3 weeks, equivalent to 100 mg/m(2) of irinotecan base) or **fluorouracil and folinic acid**. A third	Primary endpoint: OS<br><br>Safety was assessed in all patients who had received study drug.	417 (117 patients in **nanoliposomal irinotecan plus fluorouracil and folinic acid group**, 151 patients in	In **nanoliposomal irinotecan plus fluorouracil and folinic acid group**: 6.1 months<br><br>In **fluorouracil and folinic acid**	NA

					arm consisting of **nanoliposomal irinotecan (80 mg/m(2),** equivalent to 70 mg/m(2) of irinotecan base) **with fluorouracil and folinic acid** every 2 weeks was added later (1:1:1), in a protocol amendment.		**nanoliposomal irinotecan monotherapy group**, and 149 patients in **fluorouracil and folinic acid** group).	**group**: 4.2 months<br><br>In **nanoliposomal irinotecan monotherapy group**: 4.9 months.	
11	Phase II<br><br>A prospective trial which assesses outcomes of patients treated with adjuvant gemcitabine/ cisplatin, stratifying results by tumor excision repair cross-complementing group-1 (ERCC1) expression.	Postlewait *et al.* [22]	2016	Patients with resected pancreatic adenocarcinoma which was previously treated with gemcitabine alone.	**1000 mg/m² gemcitabine plus 50 mg/m² cisplatin**. Tumor ERCC1 expression was evaluated by immune-histo-chemistry and dichotomized into low or high expression.	Primary outcomes: recurrence-free survival (RFS) and OS.	22	35.5 months	NA
12	NCT01064622<br><br>Phase Ib/II	Catenacci *et al. [16]*	2015	Patients with pancreatic cancer not amenable to curative	**Vismodegib plus gemcitabine (GV)** or **gemcitabine**	Primary outcome: PFS.	106 patients in phase II (53 in	6.9 and 6.1 months for **GV** and **GP** arms,	4.0 and 2.5 months for **GV** and **GP**

	A multicenter, randomized trial and preclinical pancreatic cancer models.			therapy who had received no prior therapy for metastatic disease.	**plus placebo (GP)**.		each arm).	respectively.	arms, respectively.
13	Phase II<br><br>An open-label, single-arm study.	Wu *et al.* [24]	2015	Patients with metastatic, un-resectable pancreatic cancer whose disease had progressed on first-line gemcitabine-based therapy.<br><br>Patients were required to have an adequate performance status (ECOG 0-2) and normal hepatic and renal function prior to being enrolled.	**Lapatinib 1250 mg** PO daily 1 h before or after meals, and **capecitabine 1000 mg/m(2)** PO twice daily on days 1-14 of the 21-day cycle.	Primary endpoint: median OS<br><br>Secondary endpoints: objective response rate, PFS and the safety profile of the combination therapy.	17	5.2 months	2.6 months
14	NCT01423604<br><br>Phase II<br><br>Randomized, Double-Blind Study.	Hurwitz *et al.* [25]	2015	Patients with metastatic pancreatic cancer who had experienced treatment failure with gemcitabine.	**Ruxolitinib** (**15 mg** twice daily) **plus capecitabine** (**1,000 mg/m(2)** twice daily) or **placebo plus capecitabine**.	Primary endpoint: OS<br><br>Secondary endpoints: PFS, clinical benefit response	127 (64 in **ruxolitinib plus capecitabine group**, 63 in **placebo plus capecita**	4.5 months in **ruxolitinib plus capecitabine group**<br><br>4.3 months in **placebo plus**	NA

						, objective response rate, and safety.	**bine group**).	**capecitabine group.**	
15	Phase I/II<br><br>An open-label, multi-center, single-arm study.	Goji *et al.* [9]	2015	Patients with unresectable pancreatic cancer confined to the pancreatic region with no earlier treatment for pancreatic cancer.	**Fixed-dose-rate gemcitabine (FDR-gem) (300-400 mg/m(2), 5 mg/m(2)/min**) on days 1, 8, 22, and 29 and **60 mg/m(2)** of **S-1 orally** on days 1-14, 22-35. **A total radiation dose** of 50.4 Gy (1.8 Gy/day, 28 fractions) was delivered concurrently.	Primary endpoint in the dose escalation phase (step 1): to establish the recommended phase II dose.<br><br>Secondary objectives: severity of adverse events, PFS, and OS.	17 patients in phase II.	16.0 months	11.0 months
16	Phase II<br><br>A multicenter study.	Makielski *et al.* [26]	2015	Patients who had no more than one previous chemotherapy regimen for their pancreatic adenocarcinoma.	**Sorafenib 200 mg** orally twice daily along with **oxaliplatin 85 mg/m(2)** IV on days 1 and 15, followed by **capecitabine 2250 mg/m(2)**	Primary objective: response rate.<br><br>Secondary objectives: PFS,	24	8.1 months (range 1.5-13.6 months).	6.0 months (range 1.5-13 months).

					orally every 8 h for six doses starting on days 1 and 15 of a 28-day cycle.	OS, and safety.			
17	NCT01608841<br><br>Phase II<br><br>A single-center, randomized, open-label, prospective trial.	Wang *et al.* [13]	2015	Chemotherapy-naïve metastatic pancreatic cancer patients.	**Gemcitabine** (**1000 mg/m2** as 30 minutes infusion on days 1, 8, 15, 22, 29, 36 and 43 followed by a 1-week rest in cycle 1 and on days 1, 8, and 15 in all subsequent 4-week cycles) or **gemcitabine plus erlotinib** (gemcitabine like gemcitabine alone, erlotinib orally **100 mg** once a day).	Primary endpoint: disease control rate.	88 (44 patients in each group).	7.2 months in **gemcitabine plus erlotinib group.**<br>4.4 months in **gemcitabine alone group.**<br>In **gemcitabine group** OS was similar regardless of the presence of EGFR mutations.<br>In **gemcitabine plus erlotinib group**, patients with EGFR mutations had a significantly longer OS (8.7 months *vs.* 6.0 months).	3.8 months in **gemcitabine plus erlotinib group.**<br>2.4 months in **gemcitabine alone group.**<br>In **gemcitabine group** PFS was similar regardless of the presence of EGFR mutations.<br>In **gemcitabine plus erlotinib group**, patients with EGFR mutatio

									ns had a significantly longer PFS (5.9 months *vs.* 2.4 months).
18	NCT00923299<br><br>Phase I/II<br><br>A single-arm, non-randomized, multicenter trial.	Assenat *et al.* [31]	2015	Advanced pancreatic cancer patients after first-line gemcitabine-based chemotherapy failure.	**Weekly cetuximab** (**400mg/m²**, then **250mg/m²**). They were sequentially included in two **trastuzumab** dose levels: **3.0 or 4.0mg/kg**, then **1.5 or 2.0mg/kg/weekly**.	Primary objective: Objective response rate, safety, progression-free survival (PFS) and overall survival (OS).	39 patients in phase 2.	4.6 months	1.8 months
19	Japic CTI-090685<br><br>Phase II<br><br>A randomized, open-label, multicenter study.	Ohkawa *et al.* [32]	2015	Patients with confirmed progressive disease following the first-line treatment with a gemcitabine-based regimen.	**S-1** (**80/100/120 mg** day (-1) based on body surface area (BSA), orally, days 1-28, every 6 weeks) or **SOX** (**S-1 80/100/120 mg** day (-1) based on BSA, orally, days 1-14, **plus oxaliplatin 100 mg m(-2)**, intravenousl	Primary endpoint: PFS<br><br>Secondary endpoints: OS, time to treatment failure (TTF), response rate (RR), disease control rate (DCR),	271 (135 patients in **S-1 group**, 136 patients in **SOX group**).	6.9 months for **S-1 group.** 7.4 months for **SOX group.**	2.8 months for **S-1 group.** 3.0 months for **SOX group.**

					y, day 1, every 3 weeks).	and safety.			
20	UMIN000009118<br><br>Phase II<br><br>A randomized clinical trial.	Shimoda *et al.* [2]	2015	Patients who had undergone resection of pancreatic cancer.	Adjuvant chemotherapy with **S-1** or **gemcitabine** after resection of pancreatic cancer.	Primary endpoint: disease-free survival (DFS).	57 (29 patients in **S-1 group**, 28 patients in **gemcitabine group**).	21.5 months in **S-1 group**, 18.0 months in **gemcitabine group.**	NA
21	NCT01079702<br><br>Phase II<br><br>An open-label, single-center study.	Kordes *et al.* [27]	2015	Patients with advanced adenocarcinoma of the pancreas were enrolled. Eligible patients had a WHO performance status 0-2 and adequate hepatic and renal functions. Also patients with prior chemotherapy in the adjuvant setting or for metastatic disease were eligible.	**Capecitabine 1000 mg/m(2)** BID day 1-14 and **everolimus 10 mg** daily (**5 mg** BID) in a continuous 21-day schedule.	Primary endpoint: response rate (RR).<br><br>Secondary endpoints: PFS, OS and 1-year survival rate.	31	8.9 months	3.6 months
22	Phase II<br><br>An open-label, single-arm study.	Cho *et al.* [17]	2015	Patients with pancreaticobiliary cancers after a curative-	Two cycles of **gemcitabine** and **docetaxel** followed by	Primary endpoint: incidence of	50 (29 patients had pancreatic cancer	17 months for patients with pancreatic cancer.	NA

				intent resection and with no prior chemotherapy or radiation therapy.	**5FU-based chemoradiation**. Four weeks after completing chemoradiation, two cycles of **gemcitabine** and **docetaxel** were administered. **Gemcitabine** was given at a dose of **1000 mg/m2** as a 30-min intravenous (IV) infusion on days 1 and 8 with **docetaxel** at **35 mg/m2** IV on days 1 and 8 of a 21-day cycle for two cycles prior to radiation therapy. **5FU** was given at **225 mg/m2** per day as a continuous infusion throughout radiation starting 3 weeks after the second	severe toxicities. Secondary endpoints: disease-free survival (DFS) and OS.	and 21 patients had biliary tract or ampullary cancers).	23 months for patients with resected biliary tract cancer.	

					cycle of gemcitabine.				
23	MPACT (NCT00844649)<br><br>Phase III<br><br>An international, multicenter, open-label study.	Goldstein *et al.* [14]	2015	Patients with metastatic pancreatic cancer with no prior chemotherapy for metastatic disease.	**Nab-paclitaxel + gemcitabine** (a 30- to 40-minute intravenous infusion of *nab*-paclitaxel **125mg/m2**, followed by an infusion of gemcitabine **1000mg/m2** on days 1, 8, 15, 29, 36, and 43) or **gemcitabine alone** (**1000mg/m2** weekly for seven of eight weeks (cycle 1)).	Primary endpoint: OS.	861 (431 patients in **nab-paclitaxel plus gemcitabine arm**, 430 patients in **gemcitabine alone arm**).	8.7 months for patients in **nab-paclitaxel plus gemcitabine group**, 6.6 months for patients in **gemcitabine alone group**.	NA
24	Phase II<br><br>A randomized study.	Petrioli *et al.* [18]	2015	Patients with metastatic pancreatic cancer with no prior chemotherapy.	The treatment in GEMOXEL arm (combination of gemcitabine, oxaliplatin and capecitabine) consisted of **gemcitabine 1,000 mg/m(2)** as a 30-min intravenous infusion on days 1, 8, 15,	Primary endpoint: disease control rate (DCR).<br><br>Secondary endpoints: safety, PFS, quality of life, and OS.	67 (34 patients in **GEMOXEL group**, 33 patients in **GEM group**).	11.9 months in **GEMOXEL arm** and 7.1 months in **GEM arm.**	6.8 months in **GEMOXEL arm** and 3.7 months in **GEM arm.**

					22, **oxaliplatin 100 mg/m(2)** i.v. on day 2, and **capecitabine 1,500 mg/m(2)/day** in two divided doses on days 1-14, every 21 days (one cycle). In gemcitabine alone group, **gemcitabine** was administered weekly for seven consecutive weeks followed by 1-week rest for the first 8 weeks, and thereafter, gemcitabine was continued on days 1, 8, 15, every 28 days.				
25	NCT01146054<br><br>Phase II<br><br>A single-arm, multi-	Herman *et al.* [19]	2015	Patients with locally advanced pancreatic cancer (LAPC) with no prior abdominal	**Gemcitabine** (**1000 mg/m(2))** for up to three weeks followed by a 1-week **break and**	Primary endpoint: the rate of late (more than 3 months after	49	13.9 months	7.8 months

	institutional study.			radiotherapy. Patients with more than 3 doses of gemcitabine before stereotactic body radiotherapy (SBRT) were excluded.	**stereotactic body radiotherapy (SBRT)** (33.0 gray [Gy] in 5 fractions). After SBRT, patients continued to receive **gemcitabine** until disease progression or toxicity.	SBRT) gastritis, fistula, enteritis or ulcer of grade more than 2 and any other late grade 3 to 4 GI toxicity attributable to gemcitabine and SBRT.<br><br>Secondary endpoints: freedom from local disease progression (FFLP); acute gastritis, fistula, enteritis, or ulcer of grade more than 2 and any other acute grade 3			

						to 4 GI toxicity attributable to gemcitabine and SBRT; OS; PFS; usefulness of FDG-PET images for estimation of survival outcome; and quality of life (QoL).			
26	NCT01065870<br><br>Phase II<br><br>A prospective study.	Sherman *et al.* [20]	2015	Pancreatic adenocarcinoma patients presenting with locally advanced, un-resectable disease because of arterial or extensive venous involvement.	Patients in both arterial and venous arms were treated with 3 week cycle for 6 cycles of **GTX (neoadjuvant gemcitabine (750 mg/m2,** days 4 and 11**), docetaxel (30 mg/m2,** days 4 and 11**),** and **capecitabine (1500 mg/m2,** days 1-14)). Those with arterial	Primary endpoint: to achieve resection in at least 50% of the patients in the arterial arm.<br><br>Patients also were followed for survival and sites	45 (34 patients in **arterial arm**, 11 patients in **venous arm**).	32.5 months for all 45 patients.<br>29 months for **arterial arm.**<br>For the **venous arm**, the median overall survival has not been reached at more than 42 months.	NA

					involvement were treated with **GX/RT (gemcitabine and capecitabine/ radiation therapy)** after **chemotherapy**.	of relapse.			
27	NCT01010126<br><br>Phase II<br><br>A two-stage multicenter single-arm open-label trial.	Hobday *et al.* [33]	2015	Patients with well or moderately differentiated pancreatic neuroendocrine tumors (PNETs) and progressive disease. Prior systemic treatments for metastatic disease were permitted.	**Temsirolimus 25 mg** intravenously (IV) once per week (on days 1, 8, 15, and 22) and **bevacizumab 10 mg/kg** IV once every 2 weeks (on days 1 and 15 of a 28-day cycle).	Co-primary end points: tumor response rate and 6-month PFS.	56	34 months	13.2 months
28	RAD001 in Advanced Neuroendocrine Tumors, Third Trial (RADIANT-3)<br>Phase III<br>A prospective, double-blind, randomized, parallel-group, placebo-controlled,	Lombard-Bohas *et al.* [34]	2015	Patients with advanced, progressive, low- or intermediate-grade pancreatic neuroendocrine tumors (pNET) with no prior cytotoxic chemotherapy, immunotherapy, or radiotherapy	Patients were prospectively stratified by **prior chemotherapy use** and World Health Organization performance status and were randomly assigned (1:1) to **everolimus 10 mg/d** or **placebo**.	Primary endpoint: PFS	410 (207 in **everolimus group**, 203 in **placebo group**).	NA	For chemo-naïve patients: 11.4 months with **everolimus** and 5.4 months with **placebo.** For patients with prior

	multicenter study.			within 4 weeks before randomization.					chemotherapy: 11.0 months with **everolimus** and 3.2 months with **placebo.**
29	CESAR (Central European Society for Anticancer Drug Research-EWIV) Phase II A prospective, randomized, open-label trial.	Bergmann *et al.* [21]	2015	Patients with locally advanced, un-resectable or metastatic pancreatic ductal adenocarcinoma (PDAC) without previous chemotherapy for adjuvant or metastatic disease.	**Gemcitabine** at a dosage of **1.000mg/m(2)** d1, 8, 15 q28 versus a combination of **SUNGEM (gemcitabine plus sunitinib)** at a dosage of **GEM 1.000mg/m(2)** d1+8 and **sunitinib 50mg** p.o. d1-14, q21d.	Primary endpoint: PFS. Secondary endpoints: OS, toxicity and overall response rate (ORR).	61 (33 patients in **gemcitabine group** and 28 patients in **SUNGEM group).**	36.7 weeks for the **GEM arm** and 30.4 weeks for **SUNGEM arm.**	13.3 weeks for **GEM arm** and 11.6 weeks for **SUNGEM arm.**
30	Phase III A randomized, multicenter study.	Lee *et al. [23]*	2017	Patients with advanced pa ncreatic cancer without prior chemotherapy.	**Gemcitabine plus capecitabine** (**oral capecitabine 1660mg/m plus gemcitabine 1000mg/m** by 30-minute intravenous infusion weekly for 3 weeks followed by a	Primary endpoint: median OS Secondary endpoints: PFS, overall response rate (ORR), disease control	214 (108 in **gemcitabine plus capecitabine group** and 106 in **gemcitabine alone group**).	10.3 months in the **gemcitabine plus capecitabine group.** 7.5 months in the **gemcitabine group**.	6.2 months in the **gemcitabine plus capecitabine group.** 5.3 months in the **gemcitabine group**.

					1-week break every 4 weeks) or **gemcitabine alone** (by 30-minute intravenous infusion weekly for 3 weeks every 4 weeks).	rate, and toxicity.			
31	Phase II A randomized, multicenter, controlled trial.	Ioka *et al.* [37]	2017	Patients with gemcitabine-refractory pancreatic cancer.	**S-1** (**80-120 mg** for 14 days every 4 weeks) **plus intravenous irinotecan** (**100 mg/m** on days 1 and 15 every 4 weeks) or **oral S-1 alone** (**80-120 mg** daily for 28 days every 6 weeks).	Primary endpoint: PFS.	127	6.8 months in **irinotecan plus S-1 group**. 5.8 months in **S-1 monotherapy group**.	3.5 months in **irinotecan plus S-1 group**. 1.9 months in **S-1 monotherapy group**.
32	UMIN000009446 Phase II A multicenter study.	Satoi *et al.* [38]	2017	Chemotherapy-naïve pancreatic ductal adenocarcinoma patients with peritoneal metastasis.	**Paclitaxel** administered intravenously at **50 mg/m** and intraperitoneal at **20 mg/m** on days 1 and 8. **S-1** administered at **80 mg/m/d** for 14 consecutive days, followed by 7 days of rest.	Primary endpoint: 1-year overall survival.<br><br>Secondary endpoints: antitumor effect and safety.	33 (22 with peritoneal dissemination and 11 with positive peritoneal cytology).	16.3 months	NA

Supplementary Table 2. Quality assessment of included studies.

Author	Selection bias	Study design	Confounders	Blinding	Data collection methods	Withdrawals and dropouts	General rating
Ettrich *et al.* [35]	Strong	Weak	NA	Weak	Strong	Moderate	Weak
Ueno *et al.* [36]	Strong	Strong	Strong	Weak	Strong	Moderate	Moderate
Tai *et al.* [39]	Strong	Weak	Strong	Weak	Strong	Weak	Weak
Uesaka *et al.* [11]	Moderate	Strong	Strong	Weak	Strong	Strong	Moderate
Hammel *et al.* [12]	Moderate	Strong	Strong	Weak	Strong	Moderate	Moderate
Stein *et al.* [28]	Strong	Weak	Weak	Weak	Strong	Moderate	Weak
Ueno *et al.* [15]	Strong	Weak	Moderate	Weak	Strong	Strong	Weak
Imaoka *et al.* [10]	Strong	Strong	Weak	Weak	Strong	Strong	Weak
Belli *et al.* [29]	Strong	Weak	NA	Weak	Strong	Strong	Weak
Wang-Gillam *et al.* [30]	Strong	Moderate	Strong	Weak	Strong	Strong	Moderate
Postlewait *et al.* [22]	Strong	Weak	Strong	Weak	Strong	Moderate	Weak
Catenacci *et al.* [16]	Strong	Strong	Strong	Weak	Moderate	Moderate	Moderate
Wu *et al.* [24]	Strong	Weak	NA	Weak	Strong	Strong	Weak
Hurwitz *et al.* [25]	Strong	Moderate	Strong	Strong	Strong	Strong	Strong
Goji *et al.* [9]	Strong	Weak	Strong	Weak	Weak	Moderate	Weak
Makielski *et al.* [26]	Strong	Weak	NA	Weak	Strong	Strong	Weak
Wang *et al.* [13]	Strong	Strong	Strong	Weak	Strong	Moderate	Moderate
Assenat *et al.* [31]	Strong	Weak	NA	Weak	Strong	Strong	Weak
Ohkawa *et al.* [32]	Strong	Strong	Strong	Weak	Strong	Strong	Moderate

Shimoda *et al.* [2]	Moderate	Strong	Strong	Weak	Moderate	Strong	Moderate
Kordes *et al.* [27]	Strong	Weak	NA	Weak	Strong	Strong	Weak
Cho *et al.* [17]	Strong	Weak	Weak	Weak	Strong	Strong	Weak
Goldstein *et al.* [14]	Strong	Moderate	Strong	Weak	Strong	Strong	Moderate
Petrioli *et al.* [18]	Strong	Moderate	Strong	Weak	Strong	Strong	Moderate
Herman *et al.* [19]	Moderate	Weak	NA	Weak	Moderate	Strong	Weak
Sherman *et al.* [20]	Strong	Weak	Moderate	Weak	Strong	Moderate	Weak
Hobday *et al.* [33]	Strong	Weak	NA	Weak	Strong	Strong	Weak
Lombard-Bohas *et al.* [34]	Strong	Moderate	Strong	Strong	Strong	Strong	Strong
Bergmann *et al.* [21]	Strong	Moderate	Strong	Weak	Strong	Strong	Moderate
Lee *et al.* [23]	Strong	Strong	Strong	Weak	Strong	Moderate	Moderate
Ioka *et al.* [37]	Strong	Moderate	Strong	Weak	Strong	Strong	Moderate
Satoi *et al.* [38]	Moderate	Weak	NA	Weak	Strong	Strong	Weak
